# Advancing Health Research Impact through a Systemic Multi-Sectoral Approach: A Protocol for Introducing Reduced-Sodium Salts and Salty Condiments in Vietnam

**DOI:** 10.3390/ijerph191912937

**Published:** 2022-10-10

**Authors:** Emalie Rosewarne, Annet C. Hoek, Aliyah Palu, Kathy Trieu, Colman Taylor, Do Thi Phuong Ha, Michael Sieburg, Nicole Ide, Kent Buse, Jacqui Webster

**Affiliations:** 1 The George Institute for Global Health, The University of New South Wales, Level 5, 1 King St., Sydney, NSW 2042, Australia; 2 Health Technology Analysts, Level 1/370 Norton St., Sydney, NSW 2040, Australia; 3 National Institute of Nutrition, Vietnam. 48B Tăng Bạt Hổ Street, Phạm Đình Hổ, Hai Bà Trưng District, Hanoi 11611, Vietnam; 4 YCP Solidiance, PTE LTD, Suite 704, Satra Dong Khoi Building, 58 Dong Khoi Street, District 1, Ho Chi Minh City 700000, Vietnam; 5 Resolve to Save Lives, 85 Broad Street, Suite 1626, New York, NY 10004, USA; 6 The George Institute for Global Health, Imperial College London, London SW7 2BX, UK

**Keywords:** global health, research impact, health policy, nutrition, Vietnam, sodium reduction, salt substitutes, healthy societies

## Abstract

Better alignment between health research organisations with the needs (and interests) of key stakeholders in the health policy and research system is critical to improving research impact. The George Institute for Global Health’s ‘Healthier Societies’ program focuses on harnessing the power of governments, markets, and communities to improve population level health equity outcomes and maximise research impact. This protocol outlines a systemic multi-sectoral approach to advance health research impact globally applied to a project to reduce population salt intake in Vietnam by introducing reduced-sodium salts and salty condiments. We defined a systemic multi-sectoral approach to be a strategy that involves engaging with government, market and communities in a deliberate and joined-up way to solve a problem in which they all have a role to play. The project objectives are to: (i) produce reduced-sodium fish sauce products and test consumer acceptability; (ii) investigate the market feasibility of introducing reduced-sodium foods (salt, bot canh and fish sauce) into the Vietnamese market; (iii) estimate the cost-effectiveness of three different government strategies to support the implementation of reduced-sodium products; and (iv) develop an advocacy roadmap to maximise potential research impact. Methods will include standard quality and safety assessments, consumer sensory testing for the locally produced reduced-sodium fish sauces, market feasibility assessment (including collating market data and semi-structured interviews with stakeholders), cost-effectiveness modelling (Markov cohort model), multi-sector stakeholder engagement, and the development of a coordinated advocacy strategy using the Kotter Plus framework. Health research organisations are increasingly seeking ways to achieve greater impact with their research. Through the application of a systemic multi-sectoral approach with governments, markets and communities, this protocol provides an example of how health research projects can achieve such impact.

## 1. Introduction

Health research systems comprise people and institutions that generate knowledge to “promote, restore, and/or maintain the health status of populations” [1]. An essential component of health research systems is health (and medical) research organisations, whose core function is to produce research to improve health [1]. Activities of these organisations also include research translation, dissemination and communication to inform health policies, strategies, practices, public opinion, and support to develop new tools or applications to improve health (e.g., health technologies) [1].

Yet, health research organisations have, to date, had limited impact in addressing societies’ health needs; a large proportion of health research appears to be wasted; investments in health research may not be sufficient and/or the research is not focused on priority health problems [2,3,4,5,6,7]. Further, there is a gap between research and policy, which not only relates to translation, dissemination, and communication of research findings, but also to how research is embedded in the policy decision-making process (i.e., how can demand for evidence by policy makers and other be increased?) [8]. Hence, the question arises: how can research impact be maximised?

As providers of high-quality evidence, health and medical research organisations have a crucial role, as collaborators and catalysts, in addressing the most pressing global health and health-equity challenges [7]. These challenges include ‘protecting people from dangerous products’ (e.g., unsafe foods and unhealthy diets) and thereby contributing to healthier societies. Reducing dietary salt intake is one of the most cost-effective public health interventions to reduce the global burden of non-communicable diseases by lowering blood pressure and thereby reducing the risk of stroke and heart disease [9,10]. Globally, people are consuming around 10 grams of salt per day [11]—double the recommended maximum amount [12]. Reduced-sodium salts, where a proportion of the sodium chloride is replaced with other salty-tasting components, such as the mineral salt potassium chloride [13,14,15], are a promising intervention to advance population salt reduction globally [16]. Evidence to date shows that replacement of standard dietary salt with reduced-sodium salts can lower systolic and diastolic blood pressure in different populations [14,17,18]. Most of these trials were in countries where salt added during cooking or at the table is the main source of salt. However, reduced-sodium salts can reduce the sodium content of processed foods, as well as table salt and salty condiments, without compromising food safety or consumer acceptability [13,16,19], so this intervention could be useful in all countries.

Hyperkalemia from increased potassium intake is a concern for individuals with chronic kidney disease [14]. However, recent modelling has demonstrated that the potential population health benefits of replacing sodium with potassium far outweigh the risks [14]. Further, results from a randomized-control trial demonstrated no difference in hyperkalemic events between participants consuming reduced-sodium salts and those consuming regular table salt [20]. Furthermore, reduced-sodium salts are intended to be used in table salt, salty condiments and high-sodium processed foods. Based on evidence, individuals with chronic kidney disease are already advised to follow a low-sodium diet where possible [21,22], including avoiding adding salt to food and consuming high-sodium condiments and processed foods. Regardless, in replacing some sodium with potassium it is important that the potassium levels are clearly labeled and easy for consumers to understand [23]. 

In Vietnam, salt intake is around 10 grams per day [24,25,26]. High salt intake is responsible for 30,000 deaths each year, with a further 150,000 deaths each year from high blood pressure, which is the leading mortality risk factor [27]. In some regions, just three products—table salt, bot canh (a type of seasoning) and fish sauce—contribute to 70% of population salt intake [24]. Thus, the consumption of reduced-sodium varieties of salt and salty condiments could substantially reduce population salt intake. Pilot studies in Vietnam have so far demonstrated the efficacy of replacing 60% of sodium with potassium in table salt and bot canh to reduce blood pressure [24]. However, the acceptability, feasibility and cost-effectiveness of an intervention to reduce population salt intake in Vietnam through the use of multiple reduced-sodium salts and salty condiments (including fish sauce) has yet to be determined.

Thus, the aim of this protocol is to outline a systemic multi-sectoral approach to advance health research impact globally, applied to a project to introduce reduced-sodium salts and salty condiments in Vietnam. We defined a systemic multi-sectoral approach to be a strategy that involves engaging with government, market and communities in a deliberate and joined-up way to solve a problem in which they all have a role to play.

### Harnessing the Power of Governments, Market, and Communities to Improve Health

The George Institute for Global Health’s (TGI) ‘Healthier Societies’ program is one of three strategic pillars of the 2025 Strategy to increase our impact on the health of millions of people worldwide and improve health equity [28]. The program is striving to achieve a systemic multi-sectoral approach through its focus on harnessing the power of governments, markets, and communities to improve health and create healthier societies [29]. This approach will help to ensure the relevance of research across the health research system and facilitate uptake and adaptation of evidence by stakeholders (e.g., policy makers and advocates).

Themes within the Healthier Societies program are broadly aligned with the socio-ecological model of factors influencing health (e.g., where people live, learn, play and work) [30] and are related to key stakeholders in the health system, namely:

- Governments: increasing the impact of governments on population health by supporting evidence-based policy;
- Markets: increasing the positive impact of the commercial sector on population health through collaboration and regulation; and
- Communities: increasing the impact of communities on their own health by harnessing internal resources to demand better policies and accountability for implementing them.

The Healthier Societies program is centred around policies and interventions likely to have the greatest health impact and, where appropriate, all three themes are embedded within research programs to maximise impact. Reducing population salt intake is one such intervention.

## 2. Materials and Methods

The overall aim of the project is to conduct exploratory research to examine the acceptability, feasibility and cost-effectiveness of reduced-sodium salts and salty condiments in Vietnam; and develop an advocacy roadmap to maximise potential research impact. The project commenced in August 2019 and is expected to be completed by August 2022. The exploratory research will comprise producing and testing the consumer acceptability of reduced-sodium fish sauce products (consumer); undertaking stakeholder research and market analysis to investigate the market feasibility and political palatability of introducing reduced-sodium salt, bot canh, and fish sauce into the market (market); and conducting a cost-effectiveness analysis to understand the costs and benefits from the perspective of the Vietnamese government (government). The exploratory research will then be used to develop a series of briefs, including policy briefs, to inform a stakeholder engagement process to establish the roadmap for advocacy to influence government policy to increase the uptake of reduced-sodium salts by markets and communities.

### 2.1. Exploratory Research

#### 2.1.1. Production and Consumer Sensory Testing of Reduced-Sodium Fish Sauce

Rationale: Food choices made by consumers and communities are influenced by their physical context (e.g., food availability and accessibility), social circumstances (e.g., cultural norms) [31,32,33] and individual motives (e.g., taste, price) [34,35,36,37]. In Vietnam, salt and salty condiments are readily available, widely used in Vietnamese cuisine and the population has a known preference for salty products [24]. So, in order to successfully introduce reduced-sodium salts and salty condiments into the market, these products must also be readily available, affordable and easily accessible (see ‘Examine Feasibility and Pathways to Market’), be able to be used in the same way in cooking and at the table (i.e., as part of a meal and as a seasoning) and be acceptable to consumers by meeting their taste preferences.

Objective: To develop reduced-sodium fish sauce, test for quality and safety, and conduct three consumer sensory tests to determine consumer acceptability.

Methods: A collaboration between the National Institute of Nutrition Vietnam and a popular local fish sauce manufacturer was established in 2019 to develop reduced-sodium fish sauce based on a popular traditional brand. The main steps of traditional fish sauce production will be undertaken: (1) fermenting fish with salt; (2) storing for 12 months; (3) extracting/condensing fish sauce; and (4) testing for quality and safety. The reduced-sodium salt will be added during the extracting/condensing stage in place of regular salt. Two recipes with different levels of sodium reduction will be produced. The reduced-sodium products will then undergo standard quality and safety assessments (aligned with General Department of Standards, Metrology and Quality) of physiochemical characteristics, microbiological characteristics, and heavy metal residues, initially and over a 12-month period. Three double-blinded consumer acceptability tests will then be conducted. Firstly, a Duo-Trio Test [38] will be performed whereby trained professionals (n = 16) will receive three fish sauce samples and be asked to differentiate between generic fish sauce and the two types of reduced-sodium fish sauce. Secondly, a Quantitative Descriptive Analysis Test [39] will be conducted where a set of trained professional fish sauce analysts (n = 8) will describe 17 attributes of fish sauce samples including aftertaste, odour and flavour, appearance and taste and mouthfeel. Any differences described between regular and reduced-sodium fish sauce will then be used to improve the reduced-sodium products. Thirdly, consumer acceptability will be tested using a Hedonic Test [40]. A group of 100 consumers (18–60 years old) will taste the fish sauce samples alone and as part of a meal and rate their overall liking and preference of each sample on a 9-point hedonic scale.

Data analysis: For the quality and safety testing, the reduced-sodium fish sauce samples will be determined to be meeting or not meeting the General Department of Standards, Metrology and Quality at 3-month intervals over the 12-month period. For the three consumer acceptability tests: (1) Duo-Trio Test—The number of times the trained professionals could correctly identify the reduced-sodium fish sauce samples was compared to the minimum number of correct answers considered to be a significant difference [38]; (2) Quantitative Descriptive Analysis Test—A Principal Component Analysis will be performed to represent the space of products and attributes [39], and sensory profiles will be illustrated using radar charts; and (3) Hedonic Test—ANOVA tests will be conducted to determine if there are differences in liking, appearance, taste or mouthfeel between the regular fish sauce and reduced-sodium fish sauce samples [40].

#### 2.1.2. Examining Feasibility and Pathways to Market

Rationale: Market factors, such as food production and distribution systems, food marketing, and food quality and safety of reduced-sodium salt, will determine the availability, accessibility, affordability, and acceptability of reduced-sodium products to consumers. Vietnam is considered a developing market, with a mix of traditional and industrial food production, and in which post-liberalisation of the economy, manufacturers operate as commercial entities [41]. Thus, understanding the operations of, and collaborating with, both traditional and industrial manufacturers is vital to ensure commercial uptake and for the successful introduction of reduced-sodium salt and salty condiments into the Vietnamese market [41,42,43]. Understanding market factors can also be used to support the Government in its considerations on supporting the introduction of reduced-sodium salts.

Objective: To understand the feasibility and pathways to market for large-scale production, distribution and uptake of reduced-sodium salt and salty condiments.

Methods: The ‘market’ approach will be a combination of: (i) a compilation of relevant market data to inform the business case; and (ii) semi-structured face-to-face interviews with stakeholders including salt and fish sauce manufacturers, food retailers, and representatives of government affairs. Market data will be collected through desk research. The interviews will explore perspectives, including drivers and barriers, on the production, marketing and regulation of reduced-sodium foods. Topics will include market size and segmentation, market competitor landscape and pathways to market, consumer preferences and behaviour, and the regulatory landscape and pathways to government action on salt reduction that will impact the market (including policies that would likely increase the uptake of reduced-sodium salts and salty condiments).

Data analysis: Market data compilation and synthesis will include the market size and growth of salt and salty condiment production, total manufacturers and top manufacturers, proportion of traditional and industrial fish sauce production, price of salt and salty condiments, distribution (e.g., traditional markets, supermarkets), and applicable regulations (e.g., national food standards). The interviews will be analysed by the aforementioned topics, as well as type of stakeholder, and be used to support the market data desk research. 

#### 2.1.3. Cost-Effectiveness of Three Different Government Interventions

Rationale: Government policy, decisions, and actions (or lack thereof) pertaining to food, nutrition, and agriculture influence market operations, which flow on to consumer food choices. These may include reformulation target setting, front-of-pack labelling, nutrition education and consumer awareness campaigns, marketing promotions/restrictions, and fiscal policies [31,33]. As governments have finite resources to implement policies, the costs versus benefits obtained by different policies often play some role in how policies are formulated and whether they are adopted and implemented [44,45]. Thus, health economic impact assessments can inform government nutrition recommendations and policy or regulatory approaches [46,47].

Objective: To estimate the cost-effectiveness of three levels of government interventions for the implementation of reduced-sodium salts and salty condiments in Vietnam to inform future policy.

Methods: A Markov cohort model will be developed to evaluate the cost-effectiveness of three approaches to introduce and promote the use of reduced-sodium table salt, bot canh (a popular seasoning) and fish sauce in Vietnam. The modelled scenarios will assume that reformulated products have 60% of their sodium chloride content replaced by potassium chloride which is consistent with existing products [48]. The modelled scenarios are: (1) voluntary: no government involvement and where 30% of the target products (salt, fish sauce and bot canh) are voluntarily reformulated by food manufacturers and 5% of consumers choose reduced-sodium products; (2) subsidised: government to provide a subsidy to manufacturers producing reduced-sodium target products, accompanied by a media and communications campaign, where 50% of the targeted products available are reduced-sodium and 40% of consumers choose them; and (3) regulatory: government to introduce legislation requiring all target products to be reduced-sodium products, where 100% of manufacturers produce reduced-sodium target products and 100% of consumers consume them [48]. The model will include data on blood pressure, stroke and ischemic heart disease incidence, and mortality in the Vietnamese population, as well as data on estimated resources and programme costs, health care costs and quality of life. The population impact of the strategies over time will be estimated based on the proportion of products reformulated and the adoption of the low salt products by consumers [48].

Data analysis: The cost-effectiveness of the three strategies compared to no intervention will be evaluated. Reductions in population salt intake, strokes and cardiovascular events will be estimated. Based on this, the lifetime cost savings and health gains per person will be estimated.

### 2.2. Developing an Advocacy Roadmap

Rationale: Multi-sectoral stakeholder engagement in health research is a critical factor to increase uptake and implementation of new evidence and knowledge [49,50]. It is to the benefit of all stakeholders, including in this case researchers, industry, consumers and policy makers, to collaborate and be actively involved throughout the research cycle to continually inform the project, including in the design and implementation of the project, and the interpretation and application of the findings (e.g., policy) [49].

Objective: To develop an advocacy roadmap to engage stakeholders in discussions about reduced-sodium salts and salty condiments. This should include Government stakeholders with a view to introducing a policy to support the introduction of reduced-sodium salts and salty condiments onto the market, market stakeholders (e.g., manufacturers) with a view to producing reduced-sodium products, and consumer stakeholders with a view to increasing consumer uptake of reduced-sodium products.

Methods: A comprehensive multi-sectoral advocacy roadmap to support the introduction of reduced-sodium salts and salty condiments in Vietnam will be developed using the Kotter Plus 10-step advocacy plan—a change management strategy adapted for public health advocacy [51]. Activities will include: collating global evidence on the use of reduced-sodium salts and the evidence generated from the exploratory research and translating it into a series of evidence briefs; mapping and developing relationships with key stakeholders (including governmental bodies, industry, health research organisations, not-for-profit organisations, communication specialists and consumer representatives) and establishing a stakeholder network; developing an advocacy logic model demonstrating potential activities, outputs and outcomes; understanding pathways to policy adoption and industry and consumer uptake, communicating the vision, empowering stakeholder action by holding a stakeholder forum and undertaking one-to-one meetings.

## 3. Discussion

This study protocol presents a multi-sector approach to advance health research impact globally, which is applied to a project to introduce reduced-sodium salts and salty condiments in Vietnam. This multi-sector approach will ensure that perspectives from key stakeholders across consumers, markets, and governments, inform the research process throughout the project cycle. This will optimise the potential for reduced-sodium salts and salty condiments to be adopted as part of the government’s program to reduce population salt intake in Vietnam as well as generating lessons to support policy uptake in other countries. It is important for the government’s program to also include a consumer awareness campaign to increase consumer understanding of the need to reduce sodium intake and how to choose reduced-sodium options, and to prevent consumers performing compensatory behaviors such as adding additional table salt before consumption [52,53].

### 3.1. Collaboration and Dissemination

A project manager will act as the single point of contact to coordinate the multi-sectoral collaboration within and across the three arms of the study, conducting regular formal and informal meetings throughout the project cycle [50]. Regular collaborative meetings will be held with a core group of consumer, market, and government experts and representatives, particularly at crucial decision-making and information-sharing moments. In addition, coordination meetings and consultations are already being convened with a wider group of representatives of global and local salt reduction initiatives (including governmental bodies, health research organisations, not-for-profit organisations, communication specialists and consumer representatives). The exploratory research will be presented to a wider audience of local and international stakeholders through a multi-sectoral stakeholder forum during the development of the advocacy roadmap to discuss acceptable and feasible national-level salt reduction action.

### 3.2. Ethics

Ethical approval for the Vietnam reduced-sodium salt project was obtained from University of New South Wales Sydney, HREAP D: Biomedical Council (Project Number: HC 190540) and approved by the National Institute of Nutrition, Ministry of Health Vietnam (Project Number: 1661/QĐ-VDD).

### 3.3. Potential Practical or Operational Issues

The COVID-19 pandemic may pose delays to the research project activities, particularly the production and consumer acceptability testing of the reduced-sodium fish sauce, and stakeholder interviews. In addition, researchers at The George Institute for Global Health are located external to Vietnam. The following challenges may be encountered: language barriers, cultural differences, and an inability to travel to Vietnam during the pandemic and consequently conducting meetings and interviews online which is likely to delay project completion.

### 3.4. Potential Project Outcomes

The potential impact of this systemic multi-sectoral approach to influence government policy is unknown as this is a novel strategy. Some governments, including the UK and Ireland, have issued advice relating to reduced-sodium salts [54] but no country has yet established a regulatory or fiscal policy to support increased uptake. As such, the results of this study will be useful for informing future public health advocacy strategies for reduced-sodium salts, salt reduction and other health promotion policies. 

Based on similar research, the potential outcomes of the exploratory research can be anticipated. Firstly, it is expected that reduced-sodium fish sauce products that meet quality and safety standards can be produced using potassium-enriched reduced-sodium salts [13,14,55]. These products are likely to be acceptable to consumers as many individuals will be unable to distinguish between reduced-sodium and regular fish sauce products [14,56]. Secondly, the market feasibility assessment is expected to allow compilation of relevant market data that can inform potential pathways to government salt reduction policy and industry uptake of reduced-sodium salts [55]. Thirdly, the cost-effectiveness analysis will determine which of three levels of government intervention will be most cost-effective. Salt reduction interventions are recognized as highly cost-effective strategies for lowering blood pressure and reducing non-communicable diseases [10]. Further, a recent randomized-controlled trial has determined that reduced-sodium salt interventions are cost-saving [57]. Based on modelling of other salt reduction interventions, it is likely the regulatory approach will be most cost-effective [58]. Finally, it is anticipated that a comprehensive 10-step advocacy roadmap will be able to be generated based on knowledge and evidence from the exploratory research, as has been done for a previous salt reduction advocacy strategy in Australia [59].

## 4. Conclusions

Through the application of a systemic multi-sectoral approach with governments, markets and communities, this protocol provides an example of how global health research projects can achieve greater impact. The approach will optimise the potential for reduced-sodium salts and salty condiments to be adopted as part of the government’s program to reduce population salt intake in Vietnam.

## Data Availability

Not applicable.

## References

[1] Pang T., Sadana R., Hanney S., Bhutta Z.A., Hyder A.A., Simon J. (2003). Knowledge for better health: A conceptual framework and foundation for health research systems. Bull. World Health Organ..

[2] Chalmers I., Glasziou P. (2009). Avoidable waste in the production and reporting of research evidence. Obstet. Gynecol..

[3] Chalmers I., Bracken M.B., Djulbegovic B., Garattini S., Grant J., Gulmezoglu A.M., Howells D.W., Ioannidis J.P.A., Oliver S. (2014). How to increase value and reduce waste when research priorities are set. Lancet.

[4] Moher D., Glasziou P., Chalmers I., Nasser M., Bossuyt P.M.M., Korevaar D.A., Graham L.D., Ravaud P., Boutron I. (2016). Increasing value and reducing waste in biomedical research: Who’s listening?. Lancet.

[5] Ijsselmuiden C., Jacobs M. (2005). Health research for development: Making health research work... for everyone. Scand. J. Public Health.

[6] Luchetti M. (2014). Global Health and the 10/90 gap. Br. J. Med. Pract..

[7] World Health Organization Urgent Health Challenges for the Next Decade 2020. https://www.who.int/news-room/photo-story/photo-story-detail/urgent-health-challenges-for-the-next-decade.

[8] Buse K., Mays N., Walt G. (2012). Making Health Policy.

[9] World Health Organization (2011). From Burden to “Best Buys”: Reducing the Economic Impact of Non-Communicable Diseases in Low- and Middle-Income Countries. https://ncdalliance.org/sites/default/files/resource_files/WHO%20From%20Burden%20to%20Best%20Buys.pdf.

[10] World Health Organization (2017). Tackling NCDs: ‘Best Buys’ and Other Recommended Interventions for the Prevention and Control of Noncommunicable Diseases. https://www.who.int/ncds/management/WHO_Appendix_BestBuys.pdf.

[11] Powles J., Fahimi S., Micha R., Khatibzadeh S., Shi P., Ezzati M., E Engell R., Lim S.S., Danaei G., Mozaffarian D. (2013). Global, regional and national sodium intakes in 1990 and 2010: A systematic analysis of 24 h urinary sodium excretion and dietary surveys worldwide. BMJ Open.

[12] World Health Organization (2012). Guideline: Sodium Intake for Adults and Children. https://www.who.int/publications/i/item/9789241504836.

[13] Farrand C., MacGregor G., Campbell N.R.C., Webster J. (2019). Potential use of salt substitutes to reduce blood pressure. J. Clin. Hypertens..

[14] Greer R.C., Marklund M., Anderson C.A.M., Cobb L.K., Dalcin A.T., Henry M., Appel L.J. (2020). Potassium-Enriched Salt Substitutes as a Means to Lower Blood Pressure: Benefits and Risks. Hypertension.

[15] World Health Organization (2015). SHAKE the Salt Habit: The SHAKE Technical Package for Salt Reduction. https://www.who.int/publications/i/item/WHO-NMH-PND-16.4.

[16] Ide N., Ajenikoko A., Steele L., Cohn J., Curtis C.J., Frieden T., Cobb L. (2020). Priority Actions to Advance Population Sodium Reduction. Nutrients.

[17] China Salt Substitute Study Collaborative Group (2007). Salt substitution: A low-cost strategy for blood pressure control among rural Chinese. A randomized, controlled trial. J. Hypertens..

[18] Marklund M., Singh G., Greer R., Cudhea F., Matsushita K., Micha R., Brady T., Zhao D., Huang L., Tian M. (2020). Estimated population wide benefits and risks in China of lowering sodium through potassium enriched salt substitution: Modelling study. BMJ.

[19] World Action on Salt & Health (2021). Other Salts and Salt Substitutes. http://www.worldactiononsalt.com/less/how/other/.

[20] Neal B., Wu Y., Feng X., Zhang R., Zhang Y., Shi J., Zhang J., Tian M., Huang L., Li Z. (2021). Effect of salt substitution on cardiovascular events and death. N. Engl. J. Med..

[21] Wright J.A., Cavanaugh K.L. (2010). Dietary Sodium in Chronic kidney Disease: A Comprehensive Approach. Semin. Dial..

[22] Valtuille R. (2021). Potential novel benefits of sodium restriction in chronic kidney disease. Curr. Hypertens. Rev..

[23] Yin X., Liu H., Webster J., Trieu K., Huffman M.D., Miranda J.J., Marklund M., Wu J.H.Y., Cobb L.K., Li K.C. (2021). Vailability, formulation, labeling, and price of low-sodium salt worldwide: Environmental scan. JMIR Public Health Surveill..

[24] Do H.T.P. (2014). Hypertension in Vietnam: Prevalence, Risk Groups and Effects of Salt Substitution. Ph.D. Thesis.

[25] Jensen P.N., Bao T.Q., Huong T.T.T., Heckbert S.R., Fitzpatrick A.L., LoGerfo J.P., Le Van Ngoc T., Mokdad A.H. (2018). The association of estimated salt intake with blood pressure in a Viet Nam national survey. PLoS ONE.

[26] Ministry of Health Viet Nam (2016). National Survey on the Risk Factors of NON-Communicable Diseases (STEPS) Vietnam 2015.

[27] Institute for Health Metrics and Evaluation (IHME) (2021). GBD Compare Data Visualization. https://vizhub.healthdata.org/gbd-compare/.

[28] The George Institute for Global Health (2019). Our Strategy. https://www.georgeinstitute.org/our-strategy.

[29] Healthier Societies for Healthy Populations Group (2020). Healthier societies for healthy populations. Lancet.

[30] Bronfenbrenner U. (1979). The Ecology of Human Development: Experiments by Nature and Design.

[31] Peeters A. (2018). Obesity and the future of food policies that promote healthy diets. Nat. Rev. Endocrinol..

[32] Mork T., Lahteenmaki L., Grunert K.G. (2019). Determinants of intention to reduce salt intake and willingness to purchase salt-reduced food products: Evidence from a web survey. Appetite.

[33] Hawkes C., Smith T.G., Jewell J., Wardle J., Hammond R.A., Friel S., Thow A.M., Kain J. (2015). Smart food policies for obesity prevention. Lancet.

[34] Rozin P. (2007). Food choice: An introduction. Understanding Consumers of Food Products.

[35] Sobal J., Bisogni C.A., Devine C.M., Jastran M. (2006). A conceptual model of the food choice process over the life course. Front. Nutr. Sci..

[36] Steptoe A., Pollard T.M., Wardle J. (1995). Development of a measure of the motives underlying the selection of food: The food choice questionnaire. Appetite.

[37] Jaeger S.R., Roigard C.M., Hunter D.C., Worch T. (2021). Importance of food choice motives vary with degree of food neophobia. Appetite.

[38] International Organization for Standardization (ISO) (2017). Sensory Analysis—Methodology—Duo-Trio Test.

[39] International Organization for Standardization (ISO) (1994). Sensory Analysis—Identification and Selection of Descriptors for Establishing a Sensory Profile by a Multidimensional Approach.

[40] International Organization for Standardization (ISO) (2014). Sensory Analysis—Methodology—General Guidance for Conducting Hedonic Tests with Consumers in a Controlled Area.

[41] Nguyen H.T.H., Wood S., Wrigley N. (2013). The emerging food retail structure of Vietnam: Phases of expansion in a post-socialist environment. Int. J. Retail Distrib. Manag..

[42] YCP Solidance (2020). Market Feasibility Assessment for Low Sodium Salt, Fish Sauce, Bot Canh, and Bot Nem for Vietnam.

[43] Maruyama M., Trung L.V. (2007). Supermarkets in Vietnam: Opportunities and Obstacles. Asian Econ. J..

[44] Brunner E., Cohen D., Toon L. (2001). Cost effectiveness of cardiovascular disease prevention strategies: A perspective on EU food based dietary guidelines. Public Health Nutr..

[45] Palmer S., Raftery J. (1999). Economic Notes: Opportunity cost. BMJ.

[46] Gyles C.L., Lenoir-Wijnkoop I., Carlberg J.G., Senanayake V., Gutierrez-Ibarluzea I., Poley M.J., Dubois D., Jones P. (2012). Health economics and nutrition: A review of published evidence. Nutr. Rev..

[47] White M., Barquera S. (2020). Mexico Adopts Food Warning Labels, Why Now?. Health Syst. Reform.

[48] Taylor C., Hoek A.C., Deltetto I., Peacock A., Ha D.T.P., Sieburg M., Hoang D., Trieu K., Cobb L.K., Jan S. (2020). The cost-effectiveness of government actions to reduce sodium intake through salt substitutes in Vietnam. Arch. Public Health.

[49] Goodman M.S., Sanders Thompson V.L. (2017). The science of stakeholder engagement in research: Classification, implementation, and evaluation. Transl. Behav. Med..

[50] Huang K.Y., Kwon S.C., Cheng S., Kamboukos D., Shelley D., Brotman L.M., Kaplan S.A., Olugbenga O., Hoagwood K. (2018). Unpacking Partnership, Engagement, and Collaboration Research to Inform Implementation Strategies Development: Theoretical Frameworks and Emerging Methodologies. Front. Public Health.

[51] Moore M., Yeatman H., Pollard C. (2013). Evaluating success in public health advocacy strategies. Vietnam J. Public Health.

[52] Buttriss J.L. (2020). Why Food Reformulation and Innovation are Key to a Healthier and More Sustainable Diet. Nutr. Bull..

[53] Mozaffarian D., Angell S.Y., Lang T., Rivera J.A. (2018). Role of government policy in nutrition—Barriers to and opportunities for healthier eating. BMJ.

[54] Scientific Advisory Committee on Nutrition and the Committee on Toxicity (2017). Potassium-Based Sodium Replacers: Assessment of the Health Benefits and Risks of Using Potassium Based Sodium Replacers in Food in the UK. https://assets.publishing.service.gov.uk/government/uploads/system/uploads/attachment_data/file/660526/SACN_COT__Potassium-based_sodium_replacers.pdf.

[55] Bernabe-Ortiz A., Trieu K., Markland M., Wu J.H., Saavedra-Garcia L., Hart A.C., Yu J., Thout S.R., Miranda J.J., Webster J. (2022). The potential scalability of salt substitutes as an intervention to lower population sodium levels. Salud Pública México.

[56] Jaenke R., Barzi F., McMahon E., Webster J., Brimblecombe J. (2017). Consumer acceptance of reformulated food products: A systematic review and meta-analysis of salt-reduced foods. Crit. Rev. Food Sci. Nutr..

[57] Li K.-C., Huang L., Tian M., Di Tanna G.L., Yu J., Zhang X., Yin X., Liu Y., Hao Z., Zhou B. (2022). Cost-Effectiveness of a Household Salt Substitution Intervention: Findings From 20 995 Participants of the Salt Substitute and Stroke Study. Circulation.

[58] Cobiac L.J., Vos T., Veerman J.L. (2010). Cost-effectiveness of interventions to reduce dietary salt intake. Heart.

[59] Rosewarne E., Moore M., Chislett W.-K., Jones A., Trieu K., Webster J. (2021). An evaluation of the Victorian Salt Reduction Partnership’s advocacy strategy for policy change. Health Res. Policy Syst..

